# Determination of Genetic Structure and Signatures of Selection in Three Strains of Tanzania Shorthorn Zebu, Boran and Friesian Cattle by Genome-Wide SNP Analyses

**DOI:** 10.1371/journal.pone.0171088

**Published:** 2017-01-27

**Authors:** George Msalya, Eui-Soo Kim, Emmanuel L. K. Laisser, Maulilio J. Kipanyula, Esron D. Karimuribo, Lughano J. M. Kusiluka, Sebastian W. Chenyambuga, Max F. Rothschild

**Affiliations:** 1 Department of Animal, Aquaculture and Range Sciences, Sokoine University of Agriculture (SUA), Morogoro, Tanzania; 2 Department of Animal Science, Iowa State University, Ames, Iowa, United States of America; 3 Ministry of Education and Vocational Training, Inspectorate Department Eastern Zone, Morogoro, Tanzania; 4 Department of Veterinary Anatomy, SUA, Morogoro, Tanzania; 5 Department of Veterinary Medicine and Public Health, SUA, Morogoro, Tanzania; 6 Nelson Mandela African Institution of Science and Technology, Arusha, Tanzania; University of Sydney Faculty of Veterinary Science, AUSTRALIA

## Abstract

**Background:**

More than 90 percent of cattle in Tanzania belong to the indigenous Tanzania Short Horn Zebu (TSZ) population which has been classified into 12 strains based on historical evidence, morphological characteristics, and geographic distribution. However, specific genetic information of each TSZ population has been lacking and has caused difficulties in designing programs such as selection, crossbreeding, breed improvement or conservation. This study was designed to evaluate the genetic structure, assess genetic relationships, and to identify signatures of selection among cattle of Tanzania with the main goal of understanding genetic relationship, variation and uniqueness among them.

**Methodology/Principal findings:**

The Illumina *Bos indicus* SNP 80K BeadChip was used to genotype genome wide SNPs in 168 DNA samples obtained from three strains of TSZ cattle namely Maasai, Tarime and Sukuma as well as two comparative breeds; Boran and Friesian. Population structure and signatures of selection were examined using principal component analysis (PCA), admixture analysis, pairwise distances (F_ST_), integrated haplotype score (iHS), identical by state (IBS) and runs of homozygosity (ROH). There was a low level of inbreeding (F~0.01) in the TSZ population compared to the Boran and Friesian breeds. The analyses of F_ST_, IBS and admixture identified no considerable differentiation between TSZ trains. Importantly, common ancestry in Boran and TSZ were revealed based on admixture and IBD, implying gene flow between two populations. In addition, Friesian ancestry was found in Boran. A few common significant iHS were detected, which may reflect influence of recent selection in each breed or strain.

**Conclusions:**

Population admixture and selection signatures could be applied to develop conservation plan of TSZ cattle as well as future breeding programs in East African cattle.

## Introduction

The Tanzania Shorthorn Zebu (TSZ) is the major type of indigenous cattle in Tanzania and is comprised of a number of strains including Maasai, Sukuma, Tarime, Iringa Red, Mkalama Dun, Singida White, Mbulu, Gogo, Chagga, Pare, Fipa and Zanzibar. These strains of TSZ have considerable differences in terms of morphological features such as body size, coat color, horn size and orientation, adaptation to different ecological conditions (specific climatic, topographical and feed conditions) and they generally show some differences in the ability to withstand drought, heat stress as well as diseases and parasites [1, 2]. The TSZ forms 95% of the 25.8 million heads of cattle in Tanzania and represent a wide gene pool with a range of genetic attributes which have not been fully exploited due to inadequate knowledge of their genetic distinctiveness [3]. Generally, TSZ animals are characterized by slow growth rates, low mature weight and low milk yield and generally low productivity [4]. All indigenous animals are considered dual purpose and supply 95% of beef and 70% of milk consumed in Tanzania. This large contribution is mainly based on the significantly larger number of local cattle compared to the improved breeds and not production per animal [5]. The low productivity of the TSZ animals is a result of a combination of factors such as low genetic potential, poor nutrition as well as diseases and parasites.

To improve productivity, the national development strategies for milk and beef production have since the 1960s, placed emphasis on the use of European, US or Asian breeds such as Friesian, Ayrshire, and Jersey (for milk production) and Simmental and Angus (for beef) among others for crossbreeding and upgrading the local cattle. Among all breeds imported in Tanzania, the Boran cattle which belongs to the Large East African Zebu (LEAZ) population, is the recommended animal for upgrading the TSZ for meat production whereas Friesian is the most preferred breed for milk production improvement. These animals are recommended because of their superior performance (meat and milk production) of their F1 or F2 crossbreds [6, 7]. These animals have been promoted by the government and aid agencies since early 1980s and are distributed in government farms and farming households in areas where programs of crossbreeding or upgrading of the local cattle have been implemented. In terms of production not much has been done and in some areas these programs have either been abandoned or are not fully supported and it has been hard to obtain good and reliable records (personal communication with Dr George Kifaro, Department of Animal Science at Sokoine University of Agriculture, 2015). Many farmers regard the introduced exotic animals as inferior to the indigenous breeds particularly in terms of ability to withstand drought, feed shortage, heat stress and endemic diseases. Therefore, the cattle production sub-sector of Tanzania continues to be dominated by the less productive TSZ animals, which in general are poorly performing animals for improvement programs, and there has been slow adoption of high producing cross-bred animals. It is therefore absolutely necessary to find methods by which genetic improvement can be optimally and sustainably implemented without losing the adaptive traits of the TSZ animals valued by farmers.

Selection within a local population is a potential and sustainable strategy in developing countries such as Tanzania [8, 9]. This is because improvement of local populations through adequate selection can sustain local breeds and, therefore, secure conservation of farm animal genetic resources. However, implementation of improvement and conservation strategies should be aided by breed specific information. Breed characterization using molecular markers such as genome-wide microsatellites and single nucleotide polymorphism (SNPs) enable determination of genetic variation and relationships within and between populations and make it possible to genetically examine differences and determine special genomic attributes of indigenous livestock populations [10 – 12]. These analyses have been performed in the African cattle populations and in their crossbreds with European breeds [11, 13, 14]. Previous studies have attempted to genetically characterize the Tanzanian indigenous cattle; however these were limited in number and utilized either microsatellite markers or random amplified polymorphic DNA (RAPD) on small sample sizes [3, 15]. Studies to assess the level of variations using high density markers such as SNP in the indigenous cattle breeds of Tanzania are lacking.

We designed this study to carry out the first comprehensive analysis of genetic variation within and among three strains of TSZ cattle and two comparative breeds; Boran and Friesian. To arrive at our conclusions, we first estimated the inbreeding levels using runs of homozygosity (ROH) and the genomic inbreeding coefficients (F-geno). The ROH represents genomic autozygosity occurring due to mating between selected and genomically related individuals. Both ROH and F-geno can be a good measure of breeding depression and reduced fitness or measures the probability that two genes at any locus in an individual are identical by descent (IBD) from the common ancestors [16]. Secondly, we analyzed the genetic variation and SNP information using principal component analysis (PCA) and thirdly, we identified the signatures of selection in each animal group. PCA and admixture were used to examine population structure [17]. Signatures of selection are regions in the genome that have been preferentially increased in frequency and fixed in a population because of natural or artificial selection and because of their functional importance in specific processes [18]. Moreover, for the purpose of designing improvement or conservation programs within domestic cattle it is necessary to consider the history or origin, lineage, ancestry or pedigree information relevant to the population under study. For example, it may be important to consider that the local cattle populations in Tanzania possibly have the same origin as cattle in other African countries. The current classification of indigenous breeds based on historical evidence and morphological characteristics in one country may therefore not be satisfactory for the purpose of designing breed improvement and conservation programs.

## Results

### Relatedness and diversity among Tanzanian cattle: Inbreeding coefficients

The ROH based inbreeding coefficient (F-ROH) and F-geno were calculated to estimate the level of inbreeding. The F-ROH ranged from 0.005 to 0.023 in TSZ strains, and was 0.012 and 0.018 in Boran and Friesian breeds respectively “Table 1”. The mean length of ROH was less than 10Mb in the Boran and greatest in the Maasai strain (>17 Mb). Individuals lacking ROH (animals with no ROH) were present in each breed (highest in Sukuma and lowest in Boran breed). The F-geno values ranged from 0.01 to 0.025 in the TSZ animals, and showed a similar trend as the F-ROH. In all animal groups, the correlation coefficient (r) between F-ROH and F-geno was above 0.5. In addition, heterozygosity was computed to assess genetic variability in our cattle populations. There was a considerably greater difference in the measures of expected heterozygosity (*He*) and observed heterozygosity (*Ho*) in the Friesian breeds whereas the difference was smaller in TSZ strains and Boran breed “Table 1”. The effective population sizes (*Ne*) of the TSZ strains were greater than the values in Friesians. Nevertheless, *Ne* has decreased consistently during the last 1000 generations “S1 Fig”. Particularly, *Ne* was greater than 1,000 in most indigenous breeds 300 generations ago and the number was reduced to 100 or less in the contemporary Tanzanian cattle populations “Table 1”.

**Table 1:** Inbreeding coefficients and effective population size in Tanzanian cattle breeds.

Breed	Sukuma	Tarime	Maasai	Boran	Friesian
**F-**_**ROH**_ **(±SD)**	0.005±0.01	0.009±0.03	0.023±0.05	0.012±0.01	0.018±0.03
**Mean length of ROH (±SD, Mb)**	10.65±11.40	13.12±13.63	17.46±14.79	9.48±6.56	9.68±7.73
**Total number of ROH**	36	56	103	99	155
**Individuals lacking ROH (%)**	64.7	32.4	51.5	12.5	26.5
**F-geno (±SD)**	0.010±0.03*	0.013±0.04*	0.025±0.05*	-0.005±0.04*	0.188±0.12**
***r***_***(F-ROH—F-geno)***_	0.61*	0.75*	0.90*	0.56*	0.54**
**Expected heterozygosity *(He)***	0.401*	0.401*	0.401*	0.400*	0.321**
**Observed heterozygosity *(Ho)***	0.396*	0.395*	0.390*	0.403*	0.228**
***Ne (10)***	67	119	96	115	36
***Ne (350)***	990	1249	1166	1210	795

SD: Standard deviation; Mb: Megabase; *r* = Correlation coefficient (approximate correlation) between ROH based inbreeding (F-_ROH_) and genomic based inbreeding (F-_geno_), calculated when no pedigree or accuracy of F-ROH or F-geno could not be obtained, values >0.7 indicate similarity; *Ne* = Effective population size (generation ago);

*Estimated in *Bos indicus*;

**Estimated in all breeds

### Relatedness and differentiation between Tanzanian cattle: Pairwise distances (F_ST_) and identical by state (IBS)

**Fig 1:**
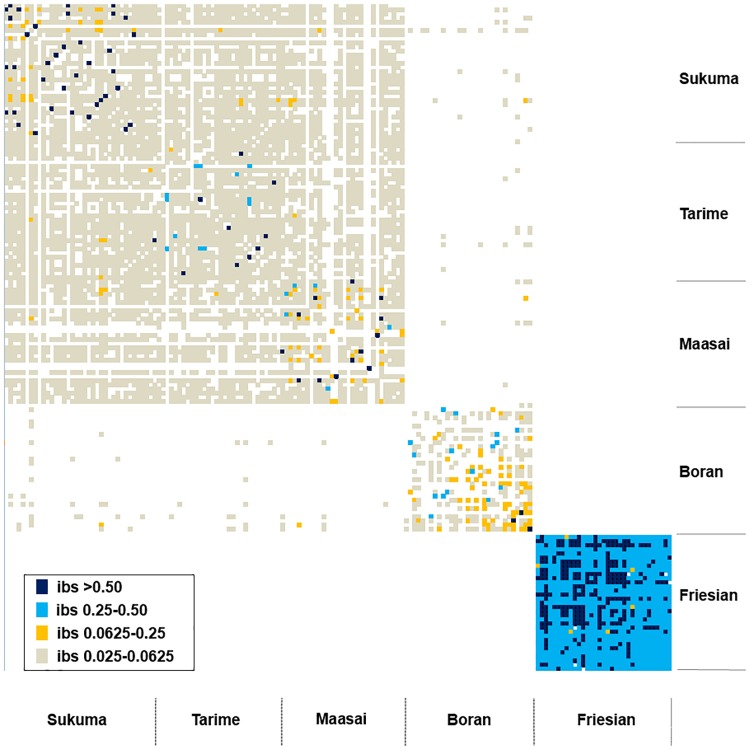
IBS between all individuals. IBS is shown with four different colours based on the range of the value. dark blue: IBS>0.5, light blue: IBS 0.25–0.5, yellow: IBS 0.0625–0.25, grey: IBS 0.025–0.0625.

The genetic variability among our animal populations and breeds was evaluated by estimation of F_ST_ and IBS “Table 2”. There was low genetic variation as reflected in the low values of F_ST_ which ranged from 0.011 to 0.013 among the TSZ strains. We also saw differences between the TSZ strains and the Boran breed (F_ST_ values ranging from 0.019 to 0.021 which were slightly larger than those obtained among the TSZ strains). When comparing TSZ strains with each other, F_ST_ was not higher than 0.4 at any loci and this result represented little difference between the strains “S2 Fig”. Between the TSZ strains and the Friesian breed F_ST_ values of approximately 0.2 were obtained, suggesting a greater differentiation. Genetic relatedness between individuals (IBS) was shown to summarize similarity between breeds “Fig 1”. The mean IBS within a TSZ strain or Boran did not exceed 0.06 “S1 Table” whereas all Friesians were related to each other (IBS > 0.25, Mean IBS = 0.41). Relatedness was observed between most individuals in different strains of TSZ. Gene flow between the Boran breed and TSZ strains was examined by IBS “Fig 1” and values were low. The analysis of the frequency of the common haplotypes showed higher correlation between TSZ strains and Boran than with Friesian “S2 Table”. Friesian was more correlated to Boran (~0.05) compared to the values between TSZ and Friesians.

**Table 2:** Differentiation (F_ST_) between Tanzanian cattle breeds/strains.

Breed/Strain	Tarime	Maasai	Boran	Friesian
**Sukuma**	0.011 (0.03)	0.013 (0.03)	0.020 (0.43)	0.202 (0.20)
**Tarime**		0.011 (0.03)	0.021 (0.43)	0.204 (0.20)
**Maasai**			0.019 (0.04)	0.208 (0.20)
**Boran**				0.186 (0.18)

Mean F_ST_ (standard deviation) between breeds/strains are shown

### Clusters, structure and admixture in Tanzanian cattle populations and breeds

**Fig 2:**
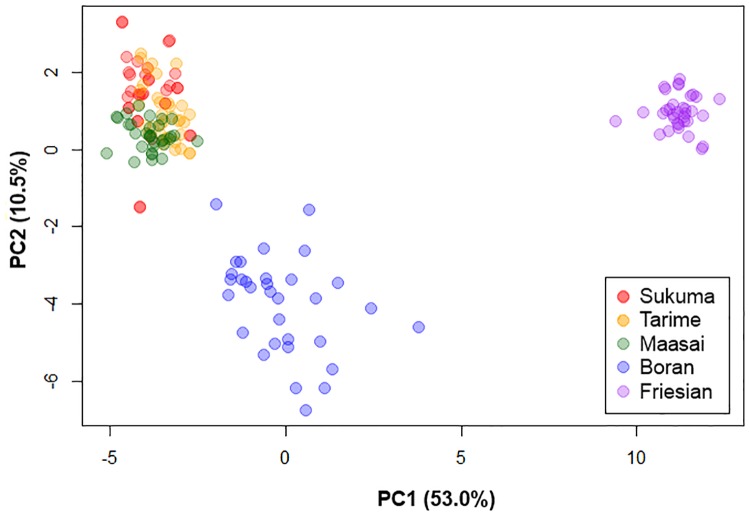
Principal components analysis between individuals of Tanzanian cattle. Sukuma (red), Tarime (yellow), Maasai (green), Boran (blue) and Friesian (purple) are indicated in different colors.

**Fig 3:**
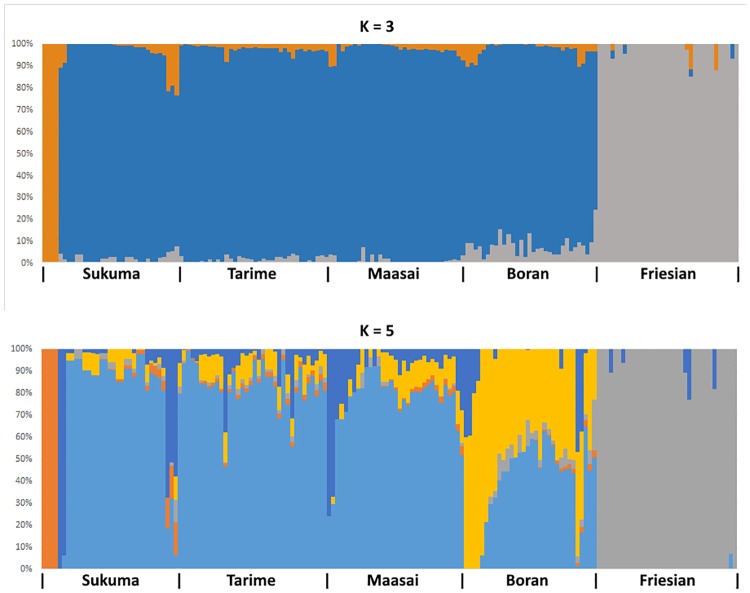
Genetic structure map in percentage probability and clustering assignment of Tanzanian cattle involved in this study. K = 3 (upper) and K = 5 (bottom) are plotted.

To further illustrate the relationship among the animals analyzed, a principle component analysis (PCA) was carried out. Animals analyzed in this study were clearly distinguishable by three clusters. Using the first principal component (PC1), the first cluster was composed of the three strains of TSZ while the second and the third clusters were composed of the Boran and Friesian breeds, respectively “Fig 2”. Regarding population/breed relationships on the second principal component (PC2) the Boran breed was shown to be genetically close to the TSZ, while the Friesian breed was slightly different from the TSZ and Boran breeds. This observation coincides with the topology of the resulting phylogenetic tree summarized in “S3 Fig” which also showed a close relationship between the TSZ populations and the Boran breed but less between the TSZ strains and the Friesian breed. Furthermore, the structure of the Tanzanian cattle analyzed in this study can be shown using a clustering assignment “Fig 3”. The TSZ animals were assumed independent strains, but no clear differentiation was identified among them. The TSZ population shared some common ancestry with Boran “Fig 3” whereas Friesians were almost unrelated to TSZ. The admixture suggested that TSZ strains could be clustered into a major and two or three minor clusters at K = 3 or K = 5. Based on the IBS, 10–15 animals in Boran appeared to be related to individuals in TSZ “Fig 1”, supporting the evidence of common ancestry among the breeds or strains. It is also noted that Boran appear to be affected by Tanzanian Friesians when considering common clusters at K = 3 or 5 “Fig 3”. The results from AMOVA supported the evidence that each of the TSZ strains were not significantly different from Boran (p>0.1) or each other (p>0.5).

### Signatures of selection in animal populations in the present study

**Fig 4:**
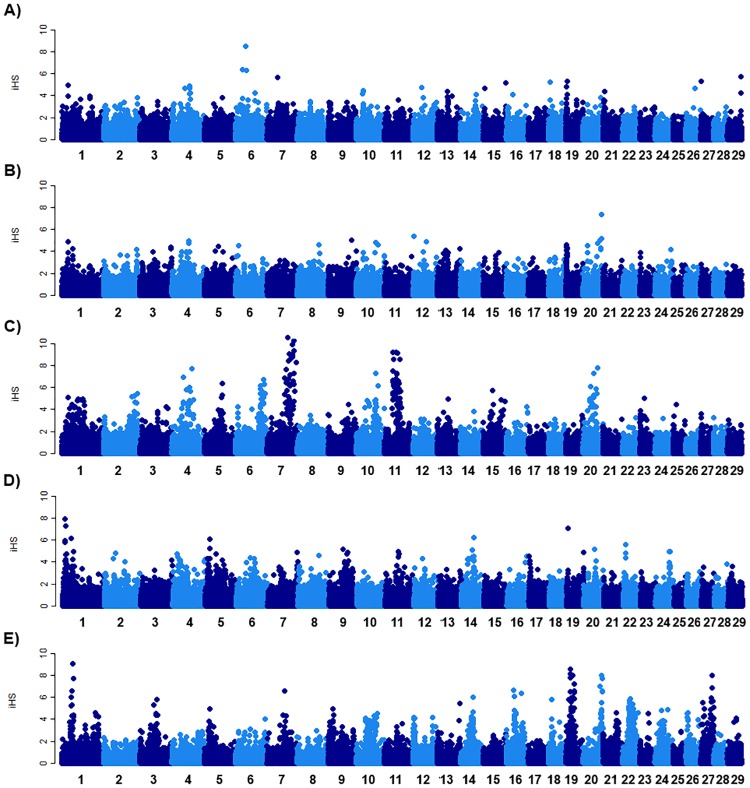
Signatures of selection (iHS) in the Tanzanian TSZ cattle population and two breeds. A: Sukuma B. Tarime C. Maasai D. Boran E. Friesian.

Some genomic regions may be fixed in individuals within a population as a result of artificial or natural selection for reasons such as adaptability or productivity. In the present study, evidence for positive selection was determined by calculating the values of iHS which measured the relative decay of extended haplotype homozygosity (EHH) of the ancestral and derived core allele. Consequently eight regions (signatures of selection) have been under recent natural or artificial selection on chromosomes 4, 5, 6, 7, 10, 11 and 20 in the Maasai strain. Three signatures of selection were detected on chromosomes 1, 5 and 14 in the Boran breed. No genomic region appeared to be involved in recent selection in the Sukuma and Tarime strains. In the Friesian breed, nine genomic regions were identified on chromosomes 1, 3, 14, 16, 19, 20, 22 and 27. A considerable number of genes were identified from the signatures of selection observed in this study. This included 20 genes in the Maasai strain, three in Boran and 19 in Friesian. Among the genes identified were the MyoD family inhibitor domain—containing gene (*MDFIC*), Stabilin 2 (*STAB2*), 5'-Nucleotidase Domain Containing 3 (*NT5DC3*), Heat shock protein 90kDa beta family member 1 (*HSP90B1*), Bovine dopamine receptor D5 (*DRD5*), Adipocyte determination and differentiation-dependent factor 1 (*ADD1*), Major facilitator superfamily domain-containing protein 10 (*MFSD10*), Small nuclear RNA activating complex polypeptide 1 (*SNAPC1*) and hypoxia inducible factor 1 alpha subunit (*HIF1A*) among others in the Maasai strain. In the Boran breed genes identified were Fibronectin type III domain containing 3B (*FNDC3B*), Solute carrier family 6 member 15 (*SLC6A15*) and Tetraspanin 19 (*TSPAN19*) genes. Whereas Gamma-aminobutyric acid type A receptor rho3 subunit (*GABRR3*), MYC induced nuclear antigen (*MINA*), cell division cycle 7 (*CDC7*), zinc finger protein 644 (*ZFP644*) and the Exostosin family 1 (*EXT1*) were among the 19 genes annotated in the Friesian breed. The signatures of selection detected among the animals in this study are presented in “Table 3” and illustrated in “Fig 4”. The genes annoted in each of the identified region are also presented in “Table 3”.

**Table 3:** Signatures of selection in Tanzanian shorthorn Zebu cattle and Boran breed.

Breed	Chr	Region (Mb)^1^	-log_10_(p)>3^2^	iHS (position)^3^	Genes^4^
**Maasai**	4	53.4–60.2	5	5.80 (53.4 Mb)	*MDFIC*, *U4*
	5	64.8–67.8	7	6.38 (67.8 Mb)	*STAB2*, *NT5DC3*, *HSP90B1*
	6	95.8–108.4	20	6.73 (107.7 Mb)	*DRD5*, *ADD1*, *MFSD10*
	7	81.2–90.3	18	10.59 (81.2 Mb)	-
	10	72.7–77.5	8	7.27 (74.2 Mb)	*SNAPC1*, *HIF1A*, *SYT16*
	11	27.1–33.5	20	13.78 (27.5 Mb)	*SIX2*, *SIX3*, *SRBD1*
	11	41.8–45.9	22	10.86 (43.3 Mb)	*REL*, *PAPOLG*, *PUS10*
	20	34.5–40.5	10	7.26 (38.4 Mb)	*CAPSL*, *IL7R*, *SPEF2*
**Boran**	1	5.8–9.8	12	7.31 (9.6 Mb)	*FNDC3B*
	5	14.7–17.3	5	6.09 (14.7 Mb)	*SLC6A15*, *TSPAN19*
	14	48.3–49.4	7	6.19 (49.0 Mb)	-
**Friesian**	1	34.8–43.6	19	9.05 (41.7 Mb)	*GABRR3*, *MINA*, *CYBG3*
	3	52.1–59.1	7	5.32 (52.3 Mb)	*CDC7*, *HFM1*, *ZNF644*
	14	42.9–49.3	26	6.01 (48.8 Mb)	*EXT1*, *MED30*, *AARD*, *RAD21*
	16	27.5–35.9	18	6.67 (28.5 Mb)	*CNIH3*,
	19	11.9–20.1	23	8.54 (16.3 Mb)	*CCL1*,*2*,*9*,*11*
	19	23.3–26.5	7	7.97 (26.5 Mb)	*WSCD1*, *NLRP1*, *MIS12*, *DERL2*
	20	65.0–71.7	25	7.99 (68.9 Mb)	-
	22	20.5–28.5	46	5.72 (24.5 Mb)	-
	27	39.6–41.0	8	7.97 (40.8 Mb)	-

Chr: Chromosome number;

^1^-log_10_ (p-value) of iHS>3, at least 5 significant values in interval are shown;

^2^Number of significant iHS in the region;

^3^Maximum -log_10_ (p-value) of iHS in the region;

^4^Genes located within 300 kb from the maximum iHS

## Discussion

This study presents the first comprehensive analysis of the genetic structure of the native cattle of Tanzania using genome-wide SNP markers. The analyses of admixture, PCA and the phylogenic tree revealed that the TSZ strains are closely related. This was supported by lower F-ROH values in the TSZ population and in Boran breed compared to the Friesian breed, low level of genetic differentiation indicated by low pairwise F_ST_ values and lack of clear clustering as well as admixture patterns in the structure map. Based on these results, it can be concluded that most of the animals in the TSZ population are closely related and might be of similar lineage and share ancestry [19, 20]. The low level of genetic differentiation among the TSZ strains may be a result of recent separation from a common ancestral population, interbreeding among them, and absence of strong artificial selection. The clustering of the TSZ into one group and the differences with other Tanzanian breeds have been shown using the RAPD markers [3] as well as the low density microsatellite markers [15]. Therefore, the characterization of TSZ animals based on geographical locations, ecological zones or external morphological characteristics (phenotypes) as done at present is not satisfactory and groups the TSZ with high levels of admixture. TSZ strains are named after tribes keeping them such as Maasai and Sukuma or location where they are predominantly found such as Tarime. The assumption that the different names of the TSZ strains reflect distinct identity may be misleading based on our results. High levels of admixture and levels of expected heterozygosity such as the ones reported in indigenous zebu populations of Tanzania in the present study have been reported in African zebu cattle elsewhere [14, 21]. Earlier reports in Tanzania have shown that the majority of indigenous cattle genetic resources are facing genetic erosion due to various factors including lack of a controlled breeding system, continuous movements of the agro-pastoralists in search of pastures and water, breeding for disease tolerance under farmers management, communal grazing systems, lack of breed development programs and introduction of exotic breeds among other factors [1, 22]. Although there has been government emphasis to improve the local animals using superior breeds [4], neither planned breeding nor formal livestock record keeping programs exist in Tanzania making it difficult to obtain information regarding pedigrees or ancestral relationships among animals.

In addition, results of admixture, IBS, Fst, and AMOVA have shown traces of TSZ populations in the Boran and Friesian breeds and vice versa. This is probably because of the sharing of recent common ancestors due to migration or closer ancient lineages as previously observed [20]. Both TSZ and Boran belong to the East African zebu (EAZ) group which includes the short horned zebu of eastern and southern Africa. The EAZ is divided into two major subgroups comprised of the Small East African zebu (SEAZ) and the LEAZ. The TSZ belongs to the SEAZ while the Boran belongs to the LEAZ. Regarding the relationship between the Friesian and TSZ, there has been relatively low or no intermixing among these breeds. Both phenomena (intermixing or lack of intermixing) have been explained previously [10]. The aurochsen strains (*Bos primigenius*) could be the origins of both the African and the European cattle populations [20]. Probably, the major variation in them is an indication of population expansion during the domestication process [19]. The movements and domestication of cattle on the African continent has been traced [19, 20]. The mitochondrial genomes of taurine (*Bos taurus*) and input of Asian zebu genes were discovered in characteristically and morphologically distinct African breeds that were regarded as zebu [20]. In our study the influence of Friesians was almost negligible in TSZ but the introgression of Friesian alleles into Boran were identified by admixture and haplotype sharing analysis, which should be considered for the future conservation plan of Boran.

Moreover the differentiation between TSZ and Friesian animals further demonstrates that most agro-pastoralists and pastoralists in rural areas do not crossbreed their indigenous breeds with dairy breeds such as Friesian. This is probably due to the fact that in agro-pastoral and pastoral communities the TSZ breed is preferred to exotic breeds or TSZ x Friesian crosses because of the adaptive characteristics of the TSZ breed to tolerate drought, feed shortages, poor quality forages and endemic diseases. Therefore, crossbreeding with exotic breeds is not a better option for achieving the long term existence of different strains of TSZ and increased productivity, but rather selection and interbreeding among the indigenous strains as different human ethnic groups intermingle.

Finally, the signatures of selection are worth mentioning and create a desire for future evaluation in terms of animal adaptation to local environments and for implementing of population breeding improvement schemes. Based on the iHS, the function of candidate genes playing important roles in cattle and other livestock species were summarized. For example the *MDFIC* gene which has been associated with modulatory roles in immune cells or immune system capabilities [23] as well as growth and development in livestock [24]. The *HSP90B1* gene is involved in a function related to lactation [25] while the *DRD5* gene has been implicated in the regulation of feeding behavior and energy homeostasis [26, 27]. In cattle *HIF1A* is one of the factors promoting vascular endothelial growth factor-induced angiogenesis during luteal development and contributes to establishing of luteal vascularization [28]. The *FNDC3B* and *ADD1* genes were associated with economically important traits in beef cattle. The gene *FNDC3B* was associated with fat deposition [29], and insertion/deletion variants of the *ADD1*/*SREBP-1c* gene have been associated with fatty acid composition [30]. The *TSPAN19* gene was identified as one of the candidate genes affecting mastitis in dairy cattle [31]. The glycosyltransferases of *EXT1* and other exostosin family genes including *EXT2*, *EXTL1*, *EXTL2*, and *EXTL3* mediate the synthesis of the backbone of Heparan sulfate proteoglycans (the ubiquitous components of the extracellular matrix) which play important roles in tissue homeostasis [32]. These genes were more likely to be involved in the recent natural selection in a breed when considering the characteristics of iHS. In contrast to Maasai and Boran, signatures of recent selection were not identified in other TSZ strains (Sukuma, Tarime) which are being bred in different geographical regions. Thus, detailed records of contagious diseases or severe changes in climate or nutrition sources for each breed will be useful information for the further understanding of the selection.

It is also worth noting that the present classification system of Tanzania has grouped the TSZ animals into more than ten strains. Of these only three were available for this study. It is therefore our recommendation that more strains be sampled and studied using genome-wide association studies such as this. In addition, zebu cattle (*Bos indicus*) are widely spread in eastern, central and southern Africa [33, 34]. Therefore carrying out comparative evaluations with animals from other countries may shed more light on the ancestry and structure of these animals. We were satisfied with the use of the Illumina *Bos indicus* chip to analyze SNPs in cattle as it appears to solve the issues of possible biasness or errors in grouping the local animals (TSZ and Boran) into the respective groups [35]. Although this may not be the case for the Friesian breed which is classified as *Bos taurus* we presumed that both *Bos indicus* and *Bos taurus* have ancestry in *Bos primigenius* [20].

To conclude, we have reported here the genetic relatedness or diversity, structure, admixture and overall relationships among cattle of Tanzania including three strains of the local TSZ population, Boran and Friesian breeds. Our results have shown that there were low levels of genetic differentiation between TSZ strains. The Boran breed was differentiated from TSZ while gene flow between them has occurred, which was supported by the analyses of IBS, admixture, and haplotype sharing. The levels of inbreeding were relatively low in TSZ compared with Western dairy cattle [36, 37] and effective population size (*Ne*) of TSZ was larger than Tanzanian Friesians. Nonetheless *Ne* has considerably decreased not only in Friesians but in TSZ strains, representing a narrow genetic pool in contemporary EAZ. Besides, the mean length of ROH in the Maasai strain was 17 Mb which is greater than the values in Western dairy breeds [36, 37] and Friesians in Tanzania. This may reflect the mating between close relatives sharing the recent common ancestors, suggesting the necessity of controlling the levels of inbreeding in the Maasai strain and TSZ populations. Inbreeding coefficients were calculated using F-geno and were negative values in some animals, which may reflect random sampling error [38]. In Friesians, strongly negative F-geno (-0.15) was obtained when estimated within the breed, although F-geno was 0.19 in all animals and F-ROH was 0.02. This may be caused by sample contamination [38], but may be due to the set of SNPs optimized for *Bos indicus*. In the analysis of clearly distinguished groups F-ROH appear to represent a reliable estimate of inbreeding, which is less dependent on the frequencies of allele and genotypes. Implementation of selection, breeding and population improvement schemes within the local population for adaptation or productivity enhancement under local environments as opposed to crossbreeding would prevent interbreeding which poses a risk of disappearance of the uniqueness of the indigenous breeds. Therefore, the genomic information identified in our study will provide an insight for the future breeding and conservation programs of cattle in Tanzania.

## Materials and Methods

### Animals and DNA purification

**Fig 5:**
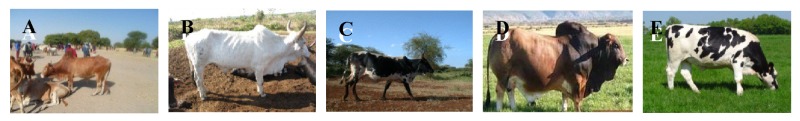
Photographs of individuals representing the Tanzania cattle populations and breeds involved in the present study. A: Sukuma B. Tarime C. Maasai D. Boran E. Friesian (Photo by ELKL).

Three strains of TSZ, namely Maasai, Tarime and Sukuma as well as two other breeds (Boran and Friesian) of cattle were involved in this study. Animals from the Maasai, Tarime and Sukuma TSZ strains were sampled from pastoralist and agro-pastoralist herds in Manyara, Mara, and Simiyu regions respectively. From each strain 40 unrelated animals were randomly sampled from four distantly (approximately 15 to 20 km apart) local villages (the smallest unit) in a region. In each village, we sampled a total of 10 animals from five herds (two animals per herd/household). The owners were asked about the relationships of the animals in order to avoid sampling of related animals. For the Boran and Friesian breeds 40 unrelated animals per breed were sampled from the government farms at Sao Hill livestock multiplication unit (LMU) and Kitulo dairy farm, respectively. In these farms, breeding records were used to avoid sampling of related animals. From all animals (represented in photographs in “Fig 5”), blood samples were obtained by jugular vein puncture using 10 ml EDTA vacutainer tubes and were immediately placed in an ice packed cool box. All blood samples were specific for the present study and were collected by experienced technicians (registered/licensed veterinarians) from the Faculty of Veterinary Medicine (FVM) at Sokoine University of Agriculture (SUA) and the methods were animal care approved. Samples were transported to the microbiology laboratory at SUA for DNA extraction within 48 hours after sampling. Blood samples were centrifuged at 2000 rpm for 20 minutes after which the plasma was discarded and buffy-coat containing peripheral blood lymphocytes was mixed with 1 ml of 8 M urea in a 2 ml cryotube.

DNA extraction followed the standard phenol-chloroform procedure [39]. Briefly, 500 μl of urea lysate was mixed with 200 μl of phenol—chloroform amyl alcohol in 1.5 ml Eppendorf tubes. The mixture was gently shaken for 2 minutes and spun at 13200 rpm for 15 minutes. To the supernatant 200 μl of 3M Sodium Acetate was added and mixed thoroughly. This mixture was spun for 15 minutes after which the resulting supernatant was utilized in the precipitation of DNA using 500 μl of ethanol. The precipitated DNA was reconstituted in 100 μl of double distilled water. Finally, agarose gel electrophoresis and optical density (OD) were performed to confirm quality of the DNA after adjusting its concentration to 50 ng/μl. All DNA samples were stored at 4°C.

### Single nucleotide polymorphism genotyping and quality control

All samples were genotyped using the GeneSeek Genomic Profiler Indicine HD Beadchip, an Illumina Infinium array consisting of 80K SNPs that were selected for the analysis of *Bos indicus* (GeneSeek, Lincoln, NE, USA). The PLINK software [38] was employed to filter out SNPs with minor allele frequency (MAF) below 0.01 and those with genotyping rate below 0.80. Also individuals (animals/samples) with more than 10% missing genotypes were excluded from further analyses. In addition, using the current bovine genome assembly of the University of Maryland (UMD) 3.1 [40], unmapped SNPs and those which were not in conformity with the Hardy-Weinberg equilibrium (P <0.0001) in each strain or breed were also excluded. In addition, only markers located on the autosomal chromosomes were selected for the diversity analysis. Therefore, out of 74,157 SNPs genotyped in 192 animals, 69,019 on autosomal chromosomes only 168 individuals (34 Sukuma zebu, 35 Tarime zebu, 32 Maasai zebu, 32 Boran and 35 Friesian) remained for further analyses.

### Estimation of genetic similarity and diversity

Two approaches were applied to calculate the inbreeding coefficient. First, the inbreeding coefficient was calculated from the sum of ROH length divided by the total length of the autosomes (genomic size) in an individual [41] and was detected using the default option (length = 1000 kb; SNPs = 100; density = 50 kb/SNP; gap = 1000 kb) of Plink *homozyg* command. The mean length of ROH was calculated from the total length of ROH divided by the total number of ROH in each breed. In addition, inbreeding levels were inferred based on the difference between observed and expected genotype frequencies (F-geno). The F-geno was obtained from the sum of single marker F using Plink *het* [38] within each breed. F-ROH is calculated from the total size of ROH in an individual which depends on the long haplotype homozygosity from the recent common ancestors and could be more sensitive to recent common ancestors compared to F-geno. To examine the changes of genetic diversity, effective population size (*Ne*) was calculated based on linkage disequilibrium (LD) in each breed/strain using the *SNeP* package [42]. To further uncover the degree of differences among the populations sampled, the extent of genetic differentiation among the populations also called pairwise distances (F_ST_) was estimated [43]. The F_ST_ was estimated using the *adegenet* package [44] in R. The genetic relatedness between animals was estimated based on the identical by state (IBS) of SNPs. *PLINK* was used for the analyses of ROH, F-geno, expected (*He*) and observed heterozygosity (*Ho*) and IBS. Haplotype was phased using *Beagle* [45]. Haplotype sharing between breeds was assessed to examine common ancestry in Tanzanian cattle. The common haplotypes were identified using 1 Mb sliding window across the genome. Then the Pearson correlation of the frequencies of the three most common haplotypes was estimated between breeds.

### Further analyses of genetic variability or diversity in Tanzania cattle by principal components and structure analyses

To ascertain the patterns of genetic diversity among Tanzanian cattle populations principal component analysis (PCA) was performed. The PCA was developed after condensation of a large number of genotypes into a few synthetic variables or clusters using the *adegenet* package in R [44]. The cross-validation option implemented by *admixture* [46] was used to estimate the most likely number (K) of underlying ancestral populations constituting a present population. The admixture analysis was performed without prior information of breed using unsupervised option. Relationship was also examined using a dendrogram constructed from allele sharing distances according to the procedure of Reynolds’ genetic distances [47] using *adegenet* [43]. For the statistical test of population structure, AMOVA was performed between breeds using the *pegas* package in R [48, 49].

### Detection of genomic regions with signatures of selection

Analyses of signatures of selection can reveal genomic regions of interest from previous selection and can be one of the powerful tools for designing a breeding program in cattle or other livestock species. In this study, the integrated haplotype score (iHS) was estimated and used to compare the EHH based on the ancestral and derived core alleles of each of the populations [50], thus enabling the detection of signatures of selection. The standardized value of iHS was computed across the genome using the *rehh* package [51]. This method requires evenly distributed SNPs across the genome, specifying derived allele for each SNP and phasing of the haplotypes. Using 80K SNPs, the density of markers was relatively high in 15 genomic regions, which may inflate the length of extended haplotype homozygosity. Considering these requirements and in order to minimize the potential of false positives, 59,390 markers were selected out of a total of 80K SNPs and considering the distance between loci (>100 kb) in the region with high density of markers (100 SNPs/1 Mb). Various regions identified as signatures of selection were considered to contain genes (regions) of importance in cattle and thus were retrieved from Biomart in Ensembl (EMBL-EBI) using Enrichr [52] or WikiPathways [53] and finally were annotated for the biological functions of those genes or genomic regions.

### Ethics and animal welfare

In Tanzania, research permits are provided by the Commission for Science and Technology (COSTECH). Permit No. SUA/ADM/R. 1/8 was issued by the SUA Vice Chancellor on behalf of COSTECH to undertake our survey and sampling in private farms in Manyara, Mara, and Simiyu regions as well as Sao Hill LMU and Kitulo dairy farms in Iringa region. In addition, permission was requested from all local authorities in the study areas whereas the verbal consent was obtained from each project participants after explaining the purpose and importance of the study prior to commencement of sampling. The decision of using the verbal consent was based on a previous experience of working with the Tanzanian farmers who understand better using either explained or visual consent (Msalya *et al*., Report of the SUA/ILRI Cow Killer Project, unpublished). Participation in the study was on a voluntary basis upon acceptance through the verbal consent which can be evidenced with their willingness to fill out our questionnaire in another study [54]. All the information collected or laboratory results obtained after the analysis of blood and DNA samples were kept under the custody of researchers as confidential. Ethics in human research in Tanzania are evaluated and permits are issued by the National Institute for Medical Research (NIMR). However, in the present study, this was not needed because no samples were needed from humans. This clarification was made by the institutional review board (IRB) of the directorate of research and postgraduate studies (DRPGS) which approves all research projects at SUA. IRB of SUA is accredited by NIMR. Specific information needed from the farmers such as identity (mainly name, gender, age, position in the household as well as household location by GPS) were collected and reported in our previous study [54]. Collection of this information was required by all 17 projects funded by The Norwegian Agency for International Development (NORAD) under the programme for Enhancing Pro-poor Innovation in Natural Resources and Agricultural Value Chains (EPINAV) and was supervised by the programme’s research and strategic intervention (RSI) component also hosted at the DRPGS at SUA. Blood samples from animals used in this study were collected humanely to safeguard well-being of animals and adhered to the Tanzanian Animal Welfare Act, 2008 (http://www.fao.org/fileadmin/user_upload/animalwelfare/tanzania.pdf). Although, there is no committee responsible for animal ethics in Tanzania, COSTECH research permits require researchers to adhere to the welfare of animals. Neither, the Institutional Animal Care and Use Committee (IACUC) was consulted as the main part of work was done in Tanzania. Laboratory analyses at GeneSeek were based on the protocols of the company (http://www.illumina.com).

## Supporting Information

S1 Fig The effective population sizes (*Ne*) of Tanzania cattle analyzed in the present study. Ne of Sukuma (dark blue), Tarime (red), Maasai (green), Boran (Purple) and Friesian (light blue) is plotted separately. X and Y axis represents generations and Ne respectively.
(TIF)

S2 Fig Genetic comparison of Tanzania cattle in this study using pairwise distances (F_ST_). The values of FST are plotted against genomic position across the genome. Comparisons between (A) Sukuma-Tarime, (B) Sukuma-Maasai, (C) Maasai-Tarime, (D) Boran-TSZ, (E) Friesian-TSZ are shown.
(TIF)

S3 Fig A Phylogenetic tree showing relationship among Tanzania cattle analyzed in this study. The distance between strains or breeds was calculated based on Reynold's method.
(TIF)

S1 Table Genetic variation (F_ST±_SD) among Tanzanian cattle. (DOCX)

S2 Table Selection signatures (iHS) in the Friesian breed. ^1^-log_10_ (p-value) of iHS>3, at least 5 significant values in interval are shown; ^2^Number of significant iHS in the region; ^3^Maximum -log10 (p-value) of iHS in the region; ^4^Genes located within 300 kb from the maximum his.
(DOCX)
