# Supplementary figures and images for: Determination of Genetic Structure and Signatures of Selection in Three Strains of Tanzania Shorthorn Zebu, Boran and Friesian Cattle by Genome-Wide SNP Analyses
Source: PLoS One. 2017 Jan 27;12(1):e0171088. doi: 10.1371/journal.pone.0171088 (PMC5271371; doi:10.1371/journal.pone.0171088)

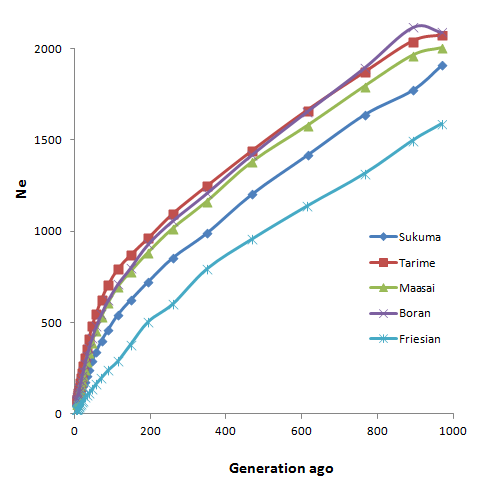

Supplement: S1 Fig — Ne of Sukuma (dark blue), Tarime (red), Maasai (green), Boran (Purple) and Friesian (light blue) is plotted separately. X and Y axis represents generations and Ne respectively. (TIF) [file pone.0171088.s001.tif]

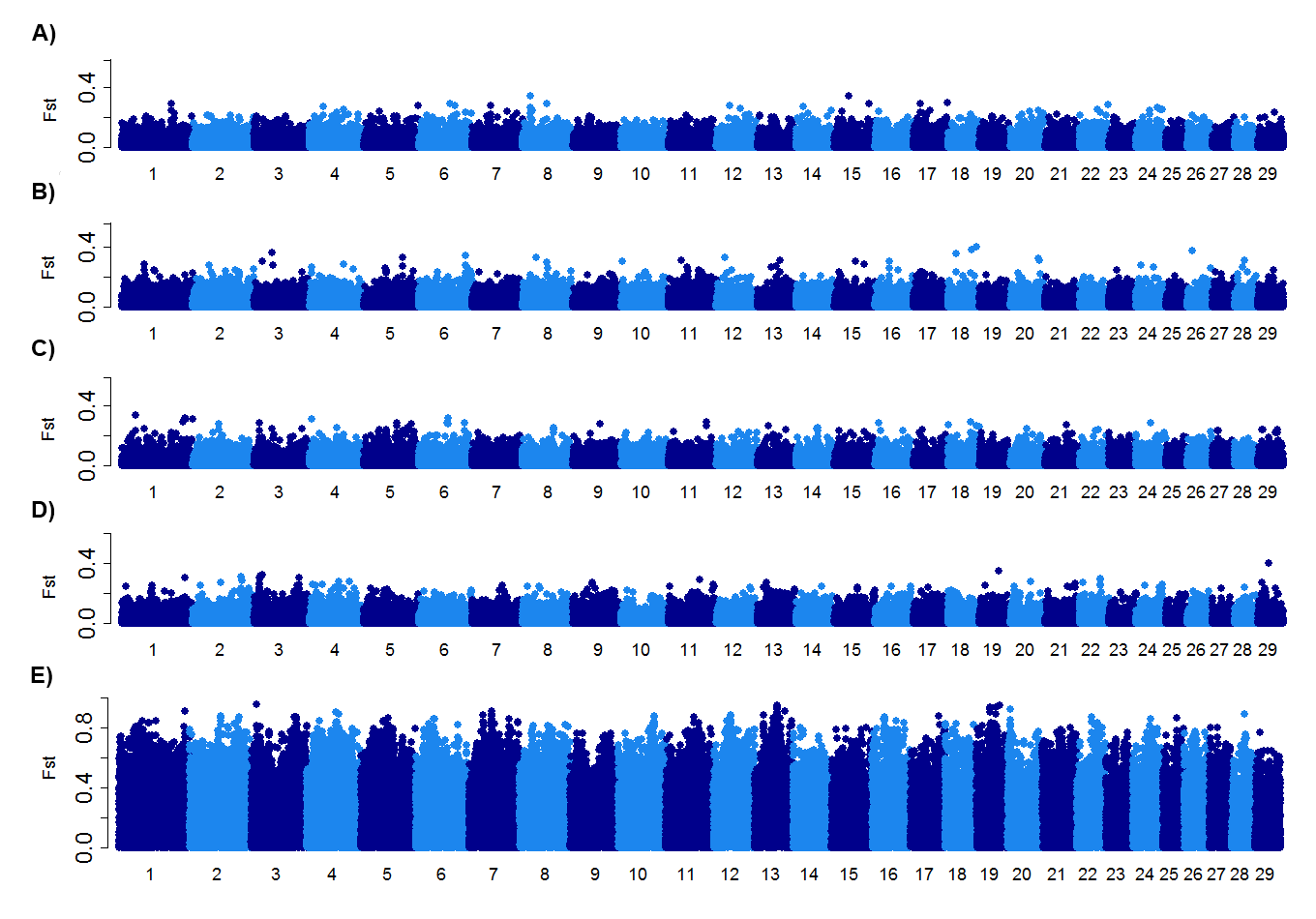

Supplement: S2 Fig — The values of FST are plotted against genomic position across the genome. Comparisons between (A) Sukuma-Tarime, (B) Sukuma-Maasai, (C) Maasai-Tarime, (D) Boran-TSZ, (E) Friesian-TSZ are shown. (TIF) [file pone.0171088.s002.tif]

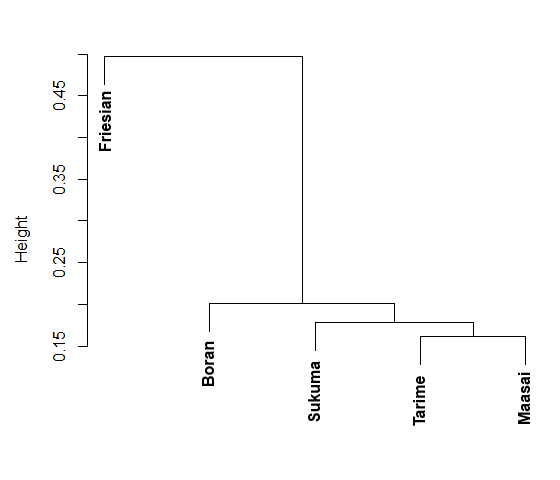

Supplement: S3 Fig — The distance between strains or breeds was calculated based on Reynold's method. (TIF) [file pone.0171088.s003.tif]
