# Supplementary material for: Determination of Genetic Structure and Signatures of Selection in Three Strains of Tanzania Shorthorn Zebu, Boran and Friesian Cattle by Genome-Wide SNP Analyses
Source: PLoS One. 2017 Jan 27;12(1):e0171088. doi: 10.1371/journal.pone.0171088 (PMC5271371; doi:10.1371/journal.pone.0171088)
Supplement: S1 Table — (DOCX) [file pone.0171088.s004.docx]

**Supporting Information**

**S1 Table. Genetic variation (F_ST±_SD) among Tanzanian cattle**

| **Breed/Ecotype** | **Tarime** | **Maasai** | **Boran** | **Friesian** |
| --- | --- | --- | --- | --- |
| **Sukuma** | 0.011±0.03 | 0.013±0.03 | 0.020±0.43 | 0.202±0.20 |
| **Tarime** |  | 0.011±0.03 | 0.021±0.43 | 0.204±0.20 |
| **Maasai** |  |  | 0.019±0.04 | 0.208±0.20 |
| **Boran** |  |  |  | 0.186±0.18 |
