# Supplementary material for: Determination of Genetic Structure and Signatures of Selection in Three Strains of Tanzania Shorthorn Zebu, Boran and Friesian Cattle by Genome-Wide SNP Analyses
Source: PLoS One. 2017 Jan 27;12(1):e0171088. doi: 10.1371/journal.pone.0171088 (PMC5271371; doi:10.1371/journal.pone.0171088)
Supplement: S2 Table — 1-log10 (p-value) of iHS>3, at least 5 significant values in interval are shown; 2Number of significant iHS in the region; 3Maximum -log10 (p-value) of iHS in the region; 4Genes located within 300 kb from the maximum his. (DOCX) [file pone.0171088.s005.docx]

**Supporting Information**

**S2 Table. Selection signatures (iHS) in the Friesian breed**

| **Chr** | **Region (Mb)^1^** | **-log_10_(p)>3^2^** | **iHS (position)^3^** | **Gene^4^** |
| --- | --- | --- | --- | --- |
| **1** | 34.8-43.6 | 19 | 9.05 (41.7 Mb) | *GABRR3, MINA, CYBG3* |
| **3** | 52.1-59.1 | 7 | 5.32 (52.3 Mb) | *CDC7, HFM1, ZNF644* |
| **14** | 42.9-49.3 | 26 | 6.01 (48.8 Mb) | *EXT1, MED30, AARD, RAD21* |
| **16** | 27.5-35.9 | 18 | 6.67 (28.5 Mb) | *CNIH3,* |
| **19** | 11.9-20.1 | 23 | 8.54 (16.3 Mb) | *CCL1,2,9,11* |
| **19** | 23.3-26.5 | 7 | 7.97 (26.5 Mb) | *WSCD1, NLRP1, MIS12, DERL2* |
| **20** | 65.0-71.7 | 25 | 7.99 (68.9 Mb) | *-* |
| **22** | 20.5-28.5 | 46 | 5.72 (24.5 Mb) | *-* |
| **27** | 39.6-41.0 | 8 | 7.97 (40.8 Mb) | *-* |

^1^-log_10_ (p-value) of iHS>3, at least 5 significant values in interval are shown; ^2^Number of significant iHS in the region; ^3^Maximum -log10 (p-value) of iHS in the region; ^4^Genes located within 300 kb from the maximum iHS
